# Parity and the risks of adverse birth outcomes: a retrospective study among Chinese

**DOI:** 10.1186/s12884-021-03718-4

**Published:** 2021-03-26

**Authors:** Li Lin, Ciyong Lu, Weiqing Chen, Chunrong Li, Vivian Yawei Guo

**Affiliations:** 1 Department of Epidemiology, School of Public Health, Sun Yat-sen University, Guangzhou, 510080 Guangdong China; 2 Chengdu Women’s and Children’s Central Hospital, School of Medicine, University of Electronic Science and Technology of China, Chengdu, 611731 Sichuan China

**Keywords:** Parity, Advanced maternal age, Preterm birth, Low birth weight, Small for gestational age

## Abstract

**Background:**

Nulliparity is considered to be a risk factor of preterm birth (PTB), low birth weight (LBW) and small for gestational age (SGA). With the new two-child policy launched in 2016, more Chinese women have delivered their 2nd baby. Yet few studies have assessed the impact of parity on adverse birth outcomes in China. This study aimed to examine the association between parity and risks of PTB, LBW and SGA in a Chinese population. The combined effects of maternal age and parity on adverse birth outcomes were also assessed.

**Methods:**

This retrospective study included all non-malformed live births born during January 1, 2014 and December 31, 2018 in Chengdu, China. A total of 746,410 eligible live singletons with complete information were included in the analysis. Parity was classified into nulliparity (i.e. has never delivered a newborn before) and multiparity (i.e. has delivered at least one newborn before). Log-binomial regression analyses were applied to evaluate the association between parity and PTB, LBW and SGA. We further divided maternal age into different groups (< 25 years, 25–29 years, 30–34 years and ≥ 35 years) to assess the combined effects of maternal age and parity on adverse birth outcomes.

**Results:**

Multiparity was associated with reduced risks of PTB (aRR = 0.91, 95% CI: 0.89–0.93), LBW (aRR = 0.74, 95% CI: 0.72–0.77) and SGA (aRR = 0.67, 95% CI: 0.66–0.69) compared with nulliparity. In each age group, we observed that multiparity was associated with lower risks of adverse birth outcomes. Compared to nulliparous women aged between 25 and 29 years, women aged ≥35 years had greater risks of PTB and LBW, regardless of their parity status. In contrast, multiparous women aged ≥35 years (aRR = 0.73, 95% CI: 0.70–0.77) and those aged < 25 years (aRR = 0.88, 95% CI: 0.84–0.93) were at lower risk of SGA compared with nulliparous women aged between 25 and 29 years.

**Conclusion:**

Multiparity was associated with lower risks of all adverse birth outcomes. Special attention should be paid to nulliparous mothers and those with advanced age during antenatal care, in order to reduce the risks of adverse birth outcomes.

**Supplementary Information:**

The online version contains supplementary material available at 10.1186/s12884-021-03718-4.

## Introduction

Preterm birth (PTB), low birth weight (LBW) and small for gestational age (SGA) are common adverse birth outcomes globally, which have become significant public health problems with increasing concerns [1–3]. Infants born with these adverse birth outcomes were at higher risks of developing neurological damage, respiratory diseases, visual and hearing impairment, as well as later life morbidity, such as stunting, mental retardation and even cerebral palsy [4–8]. Evidence has also demonstrated that PTB, LBW, SGA and its complications were main contributors to neonatal deaths and under-five mortality [9, 10]. Therefore, it is urgent to reduce the risks of adverse birth outcomes either by controlling the risk factors, or by monitoring and intervening pregnant women with high risk.

Epidemiologic evidence has demonstrated that various maternal, paternal and environmental factors, such as advanced maternal and paternal age, maternal pre-pregnant obesity, poor nutrition during pregnancy and unqualified antenatal care, might lead to adverse birth outcomes [11–14]. Parity (i.e. the number of offspring a female has delivered) was also found associated with adverse birth outcomes [15, 16]. For example, a recent study with 837,226 singleton births conducted in the Netherlands has found that the risks of PTB, very PTB and extreme PTB were significantly higher in nulliparous mothers than women who had given at least one birth before (RR: 1.95, 95%CI: 1.89–2.00 for PTB; RR: 2.15, 95%CI: 1.98–2.33 for very PTB; and RR: 2.02, 95%CI: 1.78–2.29 for extreme PTB) [17]. The increased risk of nulliparity on LBW and SGA were also demonstrated in several previous studies [15, 16, 18]. However, a systematic review consisting of 41 studies has shown that nulliparity was associated with increased risks of LBW and SGA, but not for PTB [18]. Also, significant heterogeneity was found among included studies for the outcomes of LBW and PTB [18], indicating the need for more studies.

China has altered its one-child policy to a two-child policy in 2016. With more Chinese women delivering their 2nd baby, a contemporary assessment of parity on adverse birth outcomes in Chinese women is needed. In addition, previous studies have established the “U-shaped” effects of maternal age on adverse birth outcomes [19, 20]. A meta-analysis of 14 cohort studies has found that compared to women aged between 18 and 35 years with 1–2 parity, nulliparity significantly increased the risk of PTB and infant mortality only among mothers aged less than 18 years, but not for those aged between 18 and 35 years [21]. In fact, previous studies have indicated a modification role of maternal age on the association between parity and adverse birth outcomes, including PTB, SGA and neonatal mortality [21, 22]. These findings also need confirmation in the context of China. Therefore, in the current study, we aimed to examine the association between parity and risks of PTB, LBW and SGA using a population-based sample in Chengdu, China. We further evaluated the combined effects of parity and maternal age on these three adverse birth outcomes.

## Methods

### Study design and population

A retrospective study was conducted in Chengdu, China. Data were obtained from the Chengdu Maternal and Child Health Information System, which covers information on antenatal care and birth records from all women delivering babies at any medical environment in Chengdu, China. During January 1, 2014 and December 31, 2018, 1,016,406 non-malformed live births records were extracted from the system. After excluding multiple births (*n* = 28,012, 2.76%), newborns without information of sex, height, weight and gestational age (*n* = 2257, 0.22%), as well as those without information on parity (*n* = 239,727, 23.59%), a total of 746,410 singleton live births were included in the data analysis.

The study has been approved by the institutional review board of Chengdu Women’s and Children’s Central Hospital and School of Public Health in Sun Yat-sen University. A waiver of informed consent was granted since all the retrospectively extracted data were anonymous and without any identifiable information of specific participant in our study.

### Exposure

Parity was defined as the number of children ever born to a woman. The information was self-reported by pregnant women during their first antenatal care. Women were classified into two groups: nulliparous mothers (i.e. have never delivered a newborn before) and multiparous mothers (i.e. have delivered at least one newborn before).

### Perinatal outcomes

Adverse birth outcomes in this study included PTB, LBW and SGA. Birth information was obtained from the electronic medical record system. Gestational age at birth was calculated as the period from the first day of the last menstrual cycle to the day of birth. PTB was defined as the gestational age at delivery < 37 weeks. We further divided it into moderate PTB (MPTB, gestational age between 32 and 36 weeks, *n* = 33,047) and very PTB (VPTB, gestational age < 32 weeks, *n* = 1780) for the sensitivity analyses. LBW was defined as birth weight less than 2500 g. SGA was defined as birth weight below the 10th centile for specific gestational age and sex based on the Chinese birth weight reference percentiles [23].

### Covariates

Parental age and race (Han and other ethnic minorities) were recorded during the first antenatal visit. Maternal area of residence (urban and rural), immigrant status (local residents and immigrants) and education levels (primary school or below, junior high school, senior high school and university or above) were also collected. Pre-pregnancy weight was self-reported by pregnant women during their first antenatal visit. The height was measured using a stadiometer. Pre-pregnancy body mass index (BMI) was calculated as maternal pre-pregnancy weight divided by the square of height. Maternal pre-pregnancy obesity was defined as pre-pregnancy BMI > 28 kg/m^2^ according to the standard set by the Working Group on Obesity in China [24].

### Statistical analysis

Characteristics between nulliparous and multiparous mothers was compared by independent student t-test for continuous variables and Chi-square test for categorical variables. The impact of parity on adverse birth outcomes was assessed with log-binomial regression models. In the adjusted models, parental age and race, maternal area of residence, immigrant status, education level and pre-pregnancy obesity, as well as newborn’s sex were controlled. The crude and adjusted relative risks (RRs) with 95% confidence intervals (95% CI) for PTB, LBW and SGA were calculated for multiparous mothers versus nulliparous mothers (reference), respectively. We further assessed the association between parity with MPTB and VPTB. The impact of parity on LBW and SGA was also evaluated separately in preterm and term neonates.

To evaluate the possible impact of maternal age on the association between parity and adverse birth outcomes. We first assessed the associations in different maternal age groups (i.e. < 25 years, 25–29 years, 30–34 years and ≥ 35 years). Then, the combined impact of maternal age and parity on adverse birth outcomes was evaluated, with nulliparous mothers aged between 25 and 29 years as the reference group in the multivariate log-binomial regression analyses.

To graphically visualize the birth weight between neonates born to nulliparous and multiparous mothers at different gestational age, we constructed gestational age and sex-specific birth weight charts by parity (nulliparity and multiparity) based on Box-Cox power exponential (BCPE) method [25] using generalized additive model for location, scale and shape (GAMLSS) package in R [26]. The curves of birth weight (grams) for gestational age (weeks) were smoothed by cubic splines. The best model was determined by the Akaike Information Criteria (AIC), worm plots and quantile-quantile plot.

All statistical analyses were performed with R software Version 3.6.1 (R Development Core Team). We conducted 2-sided tests with alpha set at 5%.

## Results

After excluding 241,984 women with missing data, 746,410 were included in the final analysis. The comparison of excluded and included participants are presented in Additional file 1. Except for the newborn sex, significant differences were found between the excluded and included groups for all the other characteristics. Of the included women, there were more nulliparous mothers (*n* = 427,986, 57.34%) than multiparous mothers (*n* = 318,424, 42.66%). The comparison of maternal, paternal and newborn characteristics by parity was shown in Table 1. Compared to nulliparous mothers, multiparous mothers were older (30.86 ± 4.43 years for multiparous mothers and 26.46 ± 3.77 years for nulliparous mothers), and were more likely to be rural residents, non-immigrants, less educated and with larger pre-pregnancy BMI. In terms of the adverse birth outcomes, multiparous women were more likely to give birth to preterm neonates than those who were nulliparous (5.02% versus 4.40%). However, the prevalence of LBW and SGA were significantly higher in nulliparous mothers than multiparous mothers (3.20% versus 2.84% for LBW; and 7.55% versus 5.01% for SGA).

**Table 1:** Comparison of maternal, paternal, newborn characteristics and adverse birth outcomes between nulliparity and multiparity

	Total	Nulliparity	Multiparity	***P***
N (%)	746,410	427,986 (57.34)	318,424 (42.66)	
**Maternal characteristics**				
Age (mean ± SD)	28.32 ± 4.61	26.46 ± 3.77	30.86 ± 4.43	< 0.001
Race, n (%)				0.772
Han ethnicity	719,994 (97.27)	415,772 (97.28)	304,222 (97.26)	
Other ethnicities	20,204 (2.73)	11,646 (2.72)	8558 (2.74)	
Missing	6212	568	5644	
Residence, n (%)				< 0.001
Urban	243,868 (32.94)	151,730 (35.49)	92,138 (29.45)	
Rural	496,488 (67.06)	275,779 (64.51)	220,709 (70.55)	
Missing	6054	477	5577	
Immigrant, n (%)				< 0.001
Local residents	458,356 (61.93)	244,073 (57.11)	214,283 (68.50)	
Immigrants	281,811 (38.07)	183,284 (42.89)	98,527 (31.50)	
Missing	190	152	38	
Education, n (%)				< 0.001
Primary school or below	14,668 (9.58)	3928 (0.92)	10,740 (3.43)	
Junior high school	133,422 (16.62)	45,264 (10.59)	88,158 (28.18)	
Senior high school	324,707 (40.46)	187,630 (43.89)	137,077 (43.82)	
University or above	267,559 (33.34)	190,687 (44.60)	76,872 (24.57)	
Missing	6054	477	5577	
Pre-pregnancy BMI (mean ± SD)	21.59 ± 3.29	21.03 ± 3.12	22.35 ± 3.37	< 0.001
Pre-pregnancy obesity, n (%)				< 0.001
No	710,268 (95.95)	415,926 (97.30)	294,342 (94.11)	
Yes	29,968 (4.05)	11,530 (2.70)	18,438 (5.89)	
Missing	8071	1605	6466	
**Paternal characteristics**				
Age (mean ± SD)	29.87 ± 5.71	27.84 ± 4.94	32.64 ± 5.53	< 0.001
Race, n (%)				< 0.001
Han ethnicity	720,233 (97.64)	415,145 (97.52)	305,088 (97.80)	
Other ethnicities	17,436 (2.36)	10,563 (2.48)	6873 (2.20)	
Missing	8741	2278	6463	
**Newborn characteristics**				
Sex, n (%)				0.014
Male	386,918 (51.84)	221,332 (51.71)	165,586 (52.00)	
Female	359,492 (48.16)	206,654 (48.29)	152,838 (48.00)	
Gestational week at delivery (mean ± SD)	39.07 ± 1.34	39.23 ± 1.35	38.87 ± 1.30	< 0.001
Height, cm (mean ± SD)	49.73 ± 1.50	49.71 ± 1.49	49.76 ± 1.50	< 0.001
Weight, kg (mean ± SD)	3.28 ± 0.43	3.26 ± 0.43	3.31 ± 0.43	< 0.001
**Adverse birth outcomes**				
PTB, n (%)				< 0.001
Yes	34,827 (4.67)	18,832 (4.40)	15,995 (5.02)	
No	711,583 (95.33)	409,154 (95.60)	302,429 (94.98)	
LBW, n (%)				< 0.001
Yes	22,740 (3.05)	13,701 (3.20)	9039 (2.84)	
No	723,670 (96.95)	414,285 (96.80)	309,385 (97.16)	
SGA, n (%)				< 0.001
Yes	48,233 (6.46)	32,295 (7.55)	15,938 (5.01)	
No	698,177 (93.54)	395,691 (92.45)	302,486 (94.99)	

PTB was defined as gestational age < 37 weeks, LBW was defined as birth weight < 2500 g; SGA was defined as birth weight below 10th centile for specific gestational age and sex

*Abbreviations*: *PTB* Preterm Birth, *LBW* Low Birth Weight, *SGA* Small for Gestational Age, *SD* Standard Deviation

**Fig. 1:**
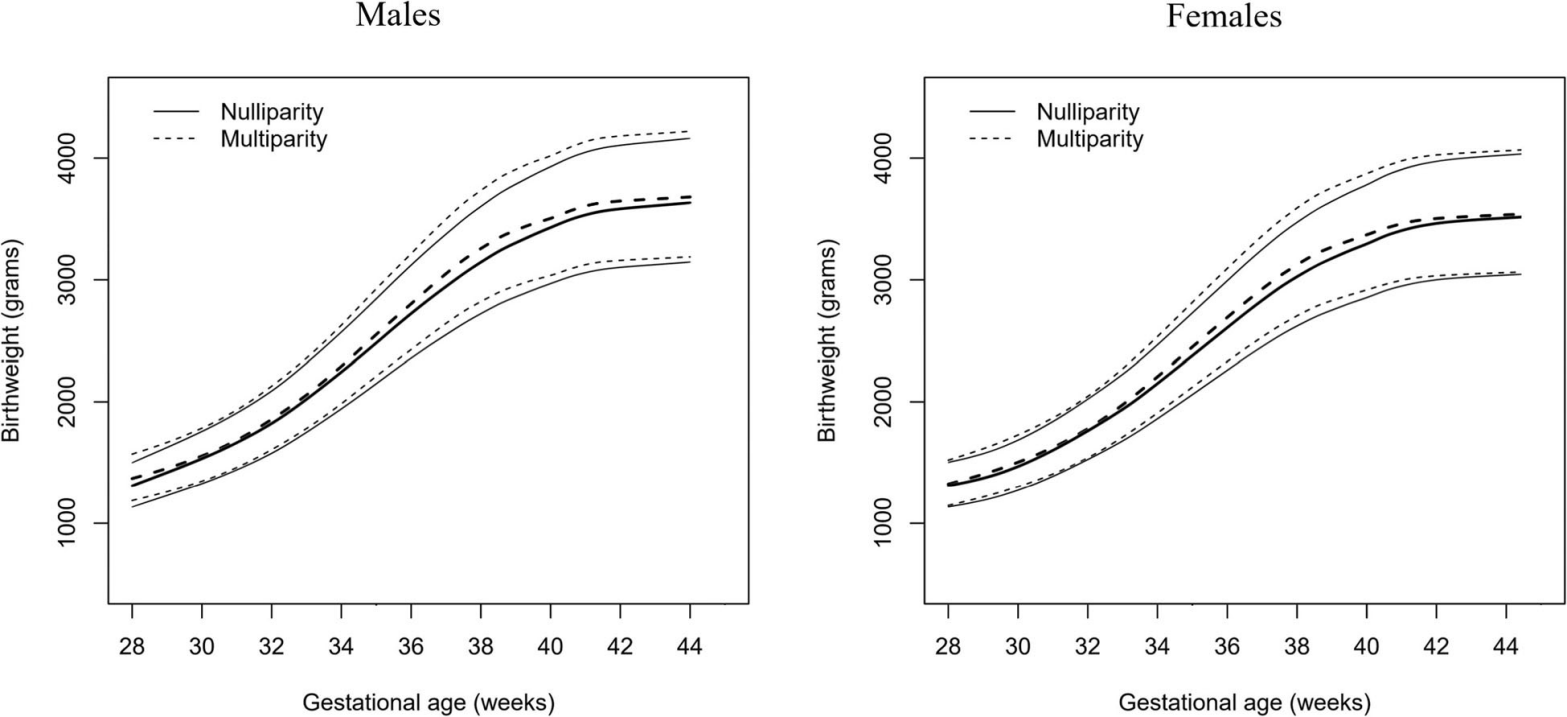
Smoothed curves of 10th, 50th and 90th percentiles of birthweight by gestational age and sex

The gestational age and sex-specific birthweight charts according to parity were shown in Fig. 1. Birthweight curves of 10th, 50th and 90th percentiles were higher for neonates born to multiparous mothers than those born to nulliparous women in both male and female newborns.

The associations between parity and adverse birth outcomes were presented in Table 2. In the univariate log-binomial regression models, the risk of PTB was increased in multiparous mothers compared with women who had never given any birth before (RR = 1.14, 95% CI: 1.12–1.17), while reduced risks of LBW (RR = 0.89,95% CI: 0.86–0.91) and SGA (RR = 0.66, 95% CI: 0.65–0.68) were found in multiparous mothers compared with nulliparous mothers. However, after adjustment for covariates, multiparity was found negatively associated with all three adverse birth outcomes (aRR = 0.91, 95% CI: 0.89–0.93 for PTB; aRR = 0.74, 95% CI: 0.72–0.77 for LBW; aRR = 0.67, 95% CI: 0.66–0.69 for SGA). In addition, parity was only statistically significant for MPTB (aRR = 0.90, 95% CI: 0.88–0.93), but not for VPTB (aRR = 1.08, 95% CI: 0.96–1.22). The significant associations between parity with LBW and SGA among both preterm and full-term newborns were consistent with our main analyses, although the magnitude of the impact of multiparity on LBW was more prominent in term babies.

**Table 2:** Relative risk for adverse birth outcomes by parity, among overall women and subgroups

	Crude RR (95% CI)	Adjusted RR (95% CI)
**PTB**		
Overall	1.14 (1.12, 1.17)	0.91 (0.89, 0.93)
MPTB	1.13 (1.11, 1.15)	0.90 (0.88, 0.93)
VPTB	1.39 (1.27, 1.53)	1.08 (0.96, 1.22)
**LBW**		
Overall	0.89 (0.86, 0.91)	0.74 (0.72, 0.77)
Preterm birth	0.89 (0.86, 0.91)	0.89 (0.86, 0.91)
Term birth	0.72 (0.69, 0.75)	0.65 (0.62, 0.69)
**SGA**		
Overall	0.66 (0.65, 0.68)	0.67 (0.66, 0.69)
Preterm birth	0.72 (0.68, 0.78)	0.68 (0.62, 0.74)
Term birth	0.66 (0.64, 0.67)	0.68 (0.66, 0.69)

MPTB was defined as gestational age between 32 and 36 weeks; VPTB was defined as gestational age between < 32 weeks; PTB was defined as gestational age < 37 weeks, LBW was defined as birth weight < 2500 g; SGA was defined as birth weight below 10th centile for specific gestational age and sex

Reference group: Nulliparity

Adjusted for maternal age and race, residence, immigrant, education, pre-pregnancy obesity, paternal age and race, sex of newborn

*Abbreviations*: *PTB* Preterm Birth, *LBW* Low Birth Weight, *SGA* Small for Gestational Age, *MPTB* Moderate Preterm Birth, *VPTB* Very Preterm Birth

**Fig. 2:**
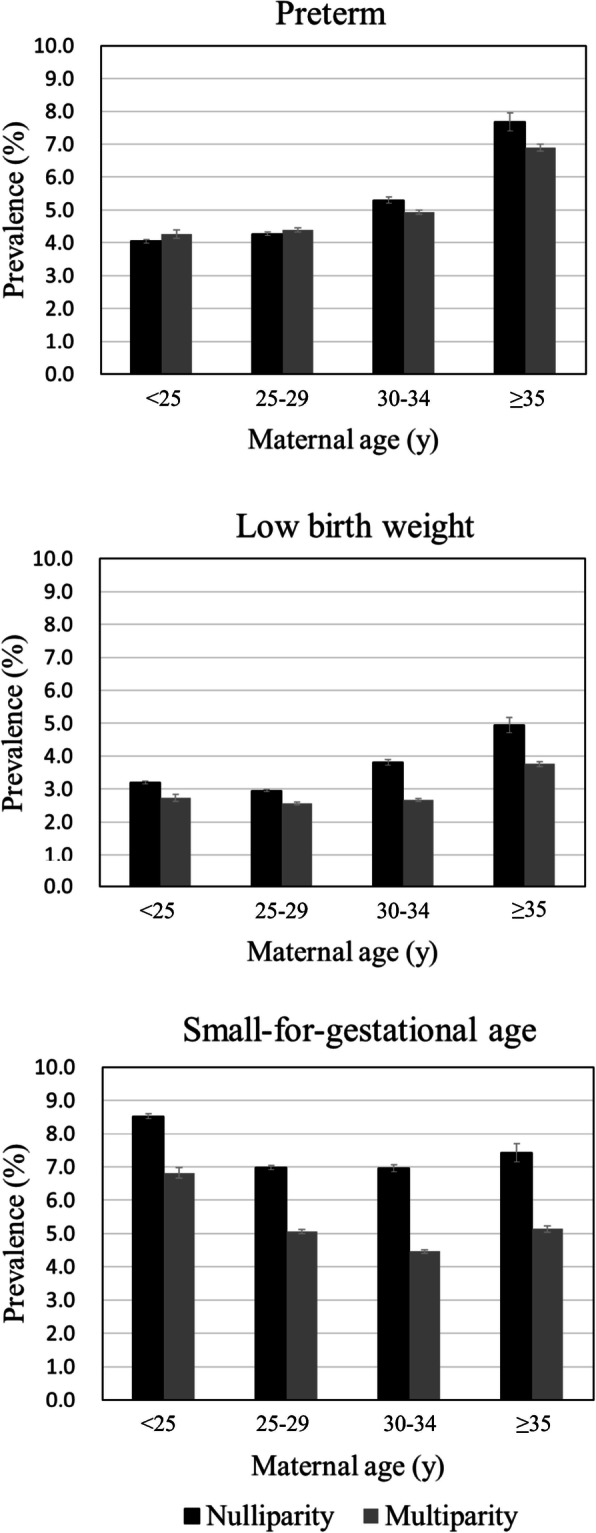
The prevalence of adverse birth outcomes by parity in each maternal age group

The prevalence of adverse birth outcomes by parity in different maternal age groups was shown in Fig. 2. The highest percentages of PTB and LBW were found in women aged ≥35 years, while women aged < 25 years were more likely to deliver a baby with SGA. Furthermore, the prevalence of adverse birth outcomes was higher among nulliparous mothers than multiparous mothers across all age groups and outcomes, except for PTB in mothers aged < 30 years. In each age groups, we assessed the impact of parity on the risks of adverse birth outcomes in both crude and adjusted models (Additional file 2). The results showed that multiparous mothers had significantly lower risks of all adverse birth outcomes across different age groups, except for PTB in women aged < 25 years (aRR = 1.02, 95% CI: 0.96–1.09). Table 3 further presents the associations between parity/maternal age and adverse birth outcomes, with nulliparous mother aged between 25 and 29 years as the reference group. We observed that nulliparous women aged ≥35 years had 63% (95% CI: 51–76%) increased risk of PTB and 56% (95% CI: 41–73%) increased risk of LBW compared with nulliparous women aged between 25 and 29 years, while the increased risk of SGA was found comparable in nulliparous women aged < 25 years (aRR = 1.14, 95% CI: 1.11–1.17) and those aged ≥35 years (aRR = 1.13, 95% CI: 1.04–1.22). In addition, multiparous mothers aged ≥35 years had increased risks of PTB (aRR = 1.39, 95% CI: 1.32–1.45) and LBW (aRR = 1.14, 95% CI: 1.07–1.21), but reduced risk of SGA (aRR = 0.73, 95% CI: 0.70–0.77) compared with the reference category.

**Table 3:** Associations between parity/maternal age and adverse birth outcomes (adjusted RR and 95% CI)

Adverse birth outcomes	Maternal age (y)			
	< 25	25–29	30–34	≥35
**PTB**				
Nulliparity	0.94 (0.90, 0.97)	1.0 (ref)	1.20 (1.15, 1.25)	1.63 (1.51, 1.76)
Multiparity	0.94 (0.88, 1.00)	0.96 (0.92, 0.99)	1.05 (1.01, 1.09)	1.39 (1.32, 1.45)
**LBW**				
Nulliparity	1.06 (1.02, 1.10)	1.0 (ref)	1.27 (1.21, 1.34)	1.56 (1.41, 1.73)
Multiparity	0.86 (0.79, 0.93)	0.81 (0.78, 0.85)	0.84 (0.80, 0.88)	1.14 (1.07, 1.21)
**SGA**				
Nulliparity	1.14 (1.11, 1.17)	1.0 (ref)	1.04 (1.01, 1.08)	1.13 (1.04, 1.22)
Multiparity	0.88 (0.84, 0.93)	0.68 (0.66, 0.70)	0.63 (0.61, 0.65)	0.73 (0.70, 0.77)

PTB was defined as gestational age < 37 weeks, LBW was defined as birth weight < 2500 g; SGA was defined as birth weight below 10th centile for specific gestational age and sex

Reference group: Nulliparity/aged between 25 and 29 years

Adjusted for maternal race, area of residence, immigrant status, education, pre-pregnancy obesity, paternal age and race, and sex of newborn

*Abbreviations*: *PTB* Preterm Birth, *LBW* Low Birth Weight, *SGA* Small for Gestational Age

## Discussion

In this retrospective study among Chinese women, birth weight for neonates born to multiparous mothers was greater than those born to nulliparous mothers, regardless of the gestational age and newborn’s sex. Compared to nulliparity, the risks of PTB, LBW and SGA in multiparous women have reduced by 9%, 26% and 33%, respectively. The association revealed in sensitivity analyses were consistent with our main findings for MPTB or in term/preterm births, except for the outcome of VPTB. We further found discrepant association between parity with PTB, LBW and SGA in different maternal age groups. Compared to nulliparous mother aged between 25 and 29 years, nulliparous women aged ≥35 years had the greatest risks of PTB and LBW, while the greatest risk of SGA was found in nulliparous women aged < 25 years and ≥ 35 years.

Our results were consistent with several previous researches [15, 17, 18, 21, 27]. For example, a retrospective cohort included 51,086 women from 2002 to 2010 found that the birthweight-for-gestational-age z-score of infants to multiparous mothers was significantly increased compared to nulliparous mothers [15]. Birthweight charts for Spanish population also demonstrated that birthweight of infants born to nulliparous women was considerably lower than those born to multiparous women [27]. Another retrospective study conducted in the Netherlands classified 802,119 women into five categories according to parity, and showed that nulliparous women had the highest risk of spontaneous PTB [17]. Nevertheless, inconsistent findings were also reported in some studies [18, 21, 28]. A meta-analysis including 41 studies suggested that nulliparity was only associated with increased risks of LBW and SGA, but not for PTB [18]. A cross-sectional study conducted in Tanzania even suggested an adverse effect of grand multiparity (parity > 5) on SGA [28]. Additionally, although the reduced risk of adverse birth outcomes related to multiparity found in our study was in line with many previous researches, the observed effect estimates were relatively small compared to some studies conducted in other populations [17, 18, 21]. For example, a retrospective study found that nulliparity was associated with nearly two-fold increased risk of PTB compared to women who had given one birth before [17]. Another meta-analysis consisting of eight studies suggested that nulliparous mothers had 89% increased risk of SGA [18]. The discrepancies might be explained by the differences in parity grouping, covariates adjusted, population characteristics and statistical models used across different studies. In our study, the majority of the multiparous mothers were delivering their 2nd babies (95.3%) due to the two-child policy in China, while several other studies included a large proportion of women who had given births for more than twice [17, 21, 28]. Inclusion of different covariates in the adjusted models might also be a source of heterogeneity. Our study additionally adjusted for paternal age and race, which were risk factors of adverse birth outcomes [29, 30], but were rarely adjusted in similar studies [17, 22]. Additionally, studies have found that the impact of maternal age and parity on adverse birth outcomes were ethnicity-specific [22]. While our study was carried out in Chinese women, the inconsistency with other studies conducted in different populations might be possible. Furthermore, most comparable studies applied logistic regression models with estimation of odds ratios (ORs) [17, 22, 31]. Since the rates of PTB, SGA and LBW were high, ORs derived from logistic regression models might cause overestimation for RR. We therefore estimated RRs directly using log-binomial regression models. Thus, the relatively smaller effect size observed in our study was plausible.

The association between parity and adverse birth outcomes might be explained by fewer uteroplacental blood flow and smaller uterine cavity of women who never gave birth before [32–37]. Studies using uterine artery doppler velocimetry found that the prevalence of uterine artery notches was significantly higher in nulliparous women compared with multiparous women, suggesting a higher uteroplacental blood impedance to flow [32, 34]. In addition, the pulsatility index of uterine artery (UtA-PI) is an indicator of vascular resistance and blood flow [38, 39]. A cross-sectional study of 1821 women showed significant increases of UtA-PI and reduction of uterine perfusion in nulliparous women, which was related to permanent uterine artery structure changes after women gave their first birth [40, 41]. Higher blood impedance and UtA-PI are related to fewer uteroplacental perfusion and blood flow, leading to insufficient nutrients and oxygen supply to fetus, which was the underlying mechanism of intrauterine growth restriction, LBW and SGA [39, 42]. Furthermore, the uterine structure of nulliparity was different from that of multiparity, where nulliparous women were more likely to have shorter endometrial cavity length and smaller uterine size [35, 43, 44]. Additionally, a retrospective study has demonstrated that women with shorter uterine length were more likely to give preterm birth [45]. Therefore, multiparous mothers could benefit from physiological changes of their former pregnancies, providing favorable conditions and basis for the growth of subsequent fetus. The negative association between multiparity and risks of adverse birth outcomes revealed in our study was biologically plausible.

We further observed a negative association between multiparity and adverse birth outcomes in every maternal age group, which were consistent with previous study [31]. Additionally, we found that both nulliparous and multiparous mothers with advanced age had increased risks of PTB and LBW compared to those who were nulliparous and aged between 25 and 29 years. Similar conclusions were also reported in previous studies [21, 22]. For example, a cohort study conducted in the US suggested that both young and advanced maternal age was associated with a higher risk of PTB compared with nulliparous women aged between 25 and 30 years, regardless of their parity status [22]. A meta-analysis synthesized data from 14 studies and showed that women with parity ≥3 and aged over 35 years had increased risks of PTB and neonatal mortality compared to women with parity 1–2 and aged between 18 and 35 years [21]. Our findings and others suggested that maternal age might play a role in the association between parity and adverse birth outcomes. It is generally acknowledged that both young and advanced maternal ages were associated with increased likelihood of adverse birth outcomes [19, 20]. In women with advanced age, the risk of obstetrical complications, such as preeclampsia, impaired placental function and decreased oocyte quality, was increased [46, 47]. These complications could subsequently increase the risks of adverse birth outcomes [48–50]. Women at young age are physiologically immature and less likely to attend appropriate antenatal care, which could also increase the risks of adverse birth outcomes, especially for teenage pregnancies [51–53].

In contrast to PTB and LBW, we found that the risk of SGA was also reduced among multiparous mothers aged ≥35 years compared with nulliparous women aged between 25 and 29 years, indicating different pathogenic mechanism of PTB, LBW and SGA. We speculated that PTB and LBW were more likely to be associated with placental and oocyte defectivity, while SGA was mainly linked to inadequacy of nutrition to fetus. However, additional studies are needed to confirm it. Additionally, due to insufficient sample size of women aged < 18 years in our study, we failed to explore the risks of adverse birth outcomes among nulliparous teenage. Further investigations are needed to figure out the potential association and mechanism.

Although parity and maternal age could not be modified, using both information might help us identify mothers with high risk of adverse birth outcomes. Previous research has shown that intervening high-risk women with targeted interventions, such as prenatal education, nutrition supplements, smoking cessation and clinical treatments during pregnancy, could effectively reduce the risk of adverse birth outcomes [54–57]. It might also be one of the most cost-effective ways to improve birth outcomes [58]. Further evidence also suggested that increasing the frequency of antenatal visits and improving the quality of antenatal care could also have positive effects on neonatal outcomes [59–61]. Therefore, specific public health and clinical strategies could be implemented for nulliparous mothers as well as women at young or advanced maternal age, to effectively improve pregnancy and neonatal outcomes.

The main strength of this retrospective study was the large sample size of singleton live births, which allowed us to conduct further subgroup and sensitivity analysis with enough power. The consistent findings in sensitivity analysis demonstrated the robustness of our study. We also plotted birthweight charts by sex of newborns in different parity categories, which enabled us to directly visualize the birthweight at different gestational age between newborns delivered by nulliparous and multiparous mothers.

Nevertheless, the present study had several limitations. Firstly, the population included in our study was limited to one city in western China and nearly one quarter (23.59%) of the study population were excluded due to missing information. Most characteristics of the included and excluded population were significantly different, which could cause potential selection bias. Cautions should be taken when extending the findings of this research to other regions of China or other countries. In particular, the majority of the included women were living in rural area (67.06%), while those excluded from this study were more likely to be urban residents (58.58%). Previous studies have shown that pregnant women in rural areas might attended fewer antenatal care with poor quality [62, 63], which subsequently could lead to adverse birth outcomes [64, 65]. However, analyses in both rural and urban mothers were in line with our main results. We further imputed the missing variables with multiple imputation and re-analyzed the data. The significant associations between parity and adverse birth outcomes were still present, suggesting the robustness of our findings (Additional files 3 and 4). Secondly, previous studies have indicated that pregnancy comorbidities (e.g. gestational hypertension and preeclampsia), maternal smoking status during pregnancy and interpregnancy interval were risks factors of adverse birth outcomes [66–69]. However, due to the unavailability of such information, we were unable to adjust these risk factors, which might result in overestimation of the effect sizes in our study. Thirdly, previous work has demonstrated that women with grand parity (i.e. women who have given births for more than five times) had increased risks of giving birth to preterm infants than women with lower parity [28], as they were usually in low socio-economic status and were more likely to have behavioral problems, such as smoking during pregnancy. Inclusion of these women in our analysis might distort the true association between parity and adverse birth outcomes. However, due to the two-child policy in China, the number of women gave five or more births was relatively small in our study (*n* = 48), which was less likely to affect the findings reported. Fourthly, although ultrasound examination might be a more reliable method to estimate gestational age [70], gestational age determined by the last-menstrual-date method was used in our study due to the lack of such information. Lastly, although previous research has shown that parity and maternal age were also associated with increased risk of still birth [71], we were unable to include such analysis due to unavailable information and therefore we might underestimate the effects of parity on adverse birth outcome.

## Conclusion

In this retrospective study, a contemporary assessment of parity on adverse birth outcomes was conducted in China. We found that nulliparity was associated with increased risks of PTB, LBW and SGA. Compared to nulliparous women aged between 25 and 29 years, mothers with advanced age had increased risks of PTB and LBW, regardless of their parity status, while multiparity was consistently associated with reduced risk of SGA across different age groups. The findings suggested that more attention should be paid to nulliparous mothers, in order to reduce the risks of adverse birth outcomes. Additionally, regardless of their parity status, women with advanced age should be closely monitored or intervened to minimize the risks of PTB and LBW.

## Supplementary Information

**Additional file 1: Table S1.** Comparison of maternal, paternal, newborn characteristics and adverse birth outcomes between excluded and included data.

**Additional file 2: Table S2.** Associations between parity and adverse birth outcomes, stratified by maternal age.

**Additional file 3: Table S3.** Relative Risk (RR) for adverse birth outcomes by parity, among overall women and subgroups with missing data imputed.

**Additional file 4: Table S4.** Associations between parity/maternal age and adverse birth outcomes with missing data imputed (adjusted RR and 95% CI).

## Data Availability

The datasets analyzed in the current study are available from the corresponding authors on reasonable request.

## Abbreviations

PTB: Preterm birth
LBW: Low birth weight
SGA: Small for gestational age
MPTB: Moderate preterm birth
VPTB: Very preterm birth
SD: Standard deviation
RR: Relative Risk
aRR: Adjusted relative risk
OR: Odds ratio
