# Supplementary material for: Parity and the risks of adverse birth outcomes: a retrospective study among Chinese
Source: BMC Pregnancy Childbirth. 2021 Mar 26;21:257. doi: 10.1186/s12884-021-03718-4 (PMC8004392; doi:10.1186/s12884-021-03718-4)
Supplement: Supplementary file 1 — Additional file 1: Table S1. Comparison of maternal, paternal, newborn characteristics and adverse birth outcomes between excluded and included data. [file 12884_2021_3718_MOESM1_ESM.docx]

**Parity and the risks of adverse birth outcomes: a retrospective study among Chinese**

Li Lin^1^, Ciyong Lu^1^, Weiqing Chen^1^, Chunrong Li ^2*^, Vivian Yawei Guo^1*^

^1^ Department of Epidemiology, School of Public Health, Sun Yat-sen University, Guangzhou, Guangdong, China

^2^ Chengdu Women's and Children's Central Hospital, School of Medicine, University of Electronic Science and Technology of China, Chengdu, Sichuan, China.

* Corresponding authors:

Chunrong Li,

Chengdu Women's and Children's Central Hospital, School of Medicine, University of Electronic Science and Technology of China, Chengdu, Sichuan, 611731, China. Email: [cdlcr@163.com](mailto:cdlcr@163.com)

Vivian Yawei Guo,

Department of Epidemiology, School of Public Health, Sun Yat-sen University, Guangzhou, Guangdong, 510080, China. Email: [guoyw23@mail.sysu.edu.cn](mailto:guoyw23@mail.sysu.edu.cn)

**Table S1** Comparison of maternal, paternal, newborn characteristics and adverse birth outcomes between excluded and included data

|  |  | **Total** | **Excluded** | **Included** | ***P*** |  |
| --- | --- | --- | --- | --- | --- | --- |
| **N (%)** | | 988394 | 241984 (24.48) | 746410 (75.52) |  |  |
| **Maternal characteristics** | | | | | |  |
| Age (mean ± SD) | | 28.54 ± 4.67 | 29.48 ± 4.83 | 28.32 ± 4.61 | < 0.001 |  |
| Race, n (%) | |  |  |  | < 0.001 |  |
|  | Han ethnicity | 883021 (96.73) | 163027 (94.39) | 719994 (97.27) |  |  |
|  | Other ethnicities | 29890 (3.27) | 9686 (5.61) | 20204 (2.73) |  |  |
|  | Missing | 75483 | 69271 | 6212 |  |  |
| Residence, n (%) | |  |  |  | < 0.001 |  |
|  | Urban | 343373 (37.60) | 99505 (57.58) | 243868 (32.94) |  |  |
|  | Rural | 569791 (62.40) | 73303 (42.42) | 496488 (67.06) |  |  |
|  | Missing | 75230 | 69176 | 6054 |  |  |
| Immigrant, n (%) | |  |  |  | < 0.001 |  |
|  | Local residents | 567191 (61.62) | 105250 (60.39) | 461941 (61.90) |  |  |
|  | Immigrants | 353320 (38.38) | 69041 (39.61) | 284279 (38.10) |  |  |
|  | Missing | 67883 | 67693 | 190 |  |  |
| Education, n (%) | |  |  |  | < 0.001 |  |
|  | Primary school or below | 18551 (2.03) | 3883 (2.25) | 14668 (1.98) |  |  |
|  | Junior high school | 150324 (16.46) | 16902 (9.78) | 133422 (18.02) |  |  |
|  | Senior high school | 388378 (42.53) | 63671 (36.84) | 324707 (43.86) |  |  |
|  | University or above | 355911 (38.98) | 88352 (51.13) | 267559 (36.14) |  |  |
|  | Missing | 75230 | 69176 | 6054 |  |  |
| Pre-pregnancy BMI (mean ± SD) | | 21.68 ± 3.27 | 22.21 ± 3.64 | 21.55 ± 3.17 | < 0.001 |  |
| Pre-pregnancy obesity, n (%) | |  |  |  | < 0.001 |  |
|  | No | 871439 (95.69) | 161783 (93.85) | 709656 (96.12) |  |  |
|  | Yes | 39286 (4.31) | 10603 (6.15) | 28683 (3.88) |  |  |
|  | Missing | 77669 | 69598 | 8071 |  |  |
| **Paternal characteristics** | | | | | |  |
| Age (mean ± SD) | | 30.23 ± 5.48 | 31.32 ± 5.67 | 29.99 ± 5.40 | < 0.001 |  |
| Race, n (%) | |  |  |  | < 0.001 |  |
|  | Han ethnicity | 882744 (97.09) | 162511 (94.76) | 720233 (97.64) |  |  |
|  | Other ethnicities | 26429 (2.91) | 8993 (5.24) | 17436 (2.36) |  |  |
|  | Missing | 79221 | 70480 | 8741 |  |  |
| **Newborn characteristics** | | | | | |  |
| Sex, n (%) | |  |  |  | 0.519 |  |
|  | Male | 512491 (51.86) | 125573 (51.91) | 386918 (51.84) |  |  |
|  | Female | 475811 (48.14) | 116319 (48.09) | 359492 (48.16) |  |  |
|  | Missing | 92 | 92 | 0 |  |  |
| Gestational week at delivery, weeks (mean ± SD) | | 39.04 ± 1.41 | 38.96 ± 1.61 | 39.07 ± 1.34 | < 0.001 |  |
|  | Missing | 1012 | 1012 | 0 |  |  |
| Height, cm (mean ± SD) | | 49.70 ± 1.64 | 49.62 ± 2.01 | 49.73 ± 1.50 | < 0.001 |  |
|  | Missing | 757 | 757 | 0 |  |  |
| Weight, kg (mean ± SD) | | 3.28 ± 0.44 | 3.27 ± 0.46 | 3.28 ± 0.43 | < 0.001 |  |
|  | Missing | 1694 | 1694 | 0 |  |  |
| **Adverse birth outcomes** | | | | | |  |
| PTB, n (%) | |  |  |  | < 0.001 |  |
|  | Yes | 50414 (5.11) | 15587 (6.47) | 34827 (4.67) |  |  |
|  | No | 936968 (94.89) | 225385 (93.53) | 711583 (95.33) |  |  |
|  | Missing | 1012 | 1012 | 0 |  |  |
| LBW, n (%) | |  |  |  | < 0.001 |  |
|  | Yes | 32842 (3.33) | 10102 (4.20) | 22740 (3.05) |  |  |
|  | No | 953858 (96.67) | 230188 (95.80) | 723670 (96.95) |  |  |
|  | Missing | 1694 | 1694 | 0 |  |  |
| SGA, n (%) | |  |  |  | < 0.001 |  |
|  | Yes | 64632 (6.55) | 16399 (6.84) | 48233 (6.46) |  |  |
|  | No | 921659 (93.45) | 223482 (93.16) | 698177 (93.54) |  |  |
|  | Missing | 2103 | 2103 | 0 |  |  |
| Abbreviation: PTB: Preterm Birth; LBW: Low Birth Weight; SGA: Small for Gestational Age; SD: Standard Deviation.  PTB was defined as gestational age < 37 weeks, LBW was defined as birth weight < 2500 g; SGA was defined as birth weight below 10th centile for specific gestational age and sex. | | | | | |  |
|  |  |  |  |  |  |  |
