# Supplementary material for: Parity and the risks of adverse birth outcomes: a retrospective study among Chinese
Source: BMC Pregnancy Childbirth. 2021 Mar 26;21:257. doi: 10.1186/s12884-021-03718-4 (PMC8004392; doi:10.1186/s12884-021-03718-4)
Supplement: Supplementary file 2 — Additional file 2: Table S2. Associations between parity and adverse birth outcomes, stratified by maternal age. [file 12884_2021_3718_MOESM2_ESM.docx]

**Parity and the risks of adverse birth outcomes: a retrospective study among Chinese**

Li Lin^1^, Ciyong Lu^1^, Weiqing Chen^1^, Chunrong Li ^2*^, Vivian Yawei Guo^1*^

^1^ Department of Epidemiology, School of Public Health, Sun Yat-sen University, Guangzhou, Guangdong, China

^2^ Chengdu Women's and Children's Central Hospital, School of Medicine, University of Electronic Science and Technology of China, Chengdu, Sichuan, China.

* Corresponding authors:

Chunrong Li,

Chengdu Women's and Children's Central Hospital, School of Medicine, University of Electronic Science and Technology of China, Chengdu, Sichuan, 611731, China. Email: [cdlcr@163.com](mailto:cdlcr@163.com)

Vivian Yawei Guo,

Department of Epidemiology, School of Public Health, Sun Yat-sen University, Guangzhou, Guangdong, 510080, China. Email: [guoyw23@mail.sysu.edu.cn](mailto:guoyw23@mail.sysu.edu.cn)

| **Table S2 Associations between parity and adverse birth outcomes, stratified by maternal age** | | | | | | | | | | | |
| --- | --- | --- | --- | --- | --- | --- | --- | --- | --- | --- | --- |
| **RR (95% CI)** | | **Maternal age (y)** | | | | | | | | | |
|  |  | **<25** | **25-29** | **30-34** | **≥35** | | | | | | |
| **PTB** | | | |  |  | | | | | | |
|  | Crude | 1.05 (0.99, 1.12) | 1.03 (0.99, 1.06) | 0.93 (0.89, 0.97) | 0.90 (0.83, 0.97) | | | | | | |
|  | Adjusted | 1.02 (0.96, 1.09) | 0.96 (0.92, 0.999) | 0.87 (0.83, 0.91) | 0.84 (0.78, 0.91) | | | | | | |
| **LBW** | | | |  |  | | | | | | |
|  | Crude | 0.85 (0.79, 0.92) | 0.87 (0.83, 0.91) | 0.70 (0.66, 0.74) | 0.76 (0.69, 0.84) | | | | | | |
|  | Adjusted | 0.81 (0.74, 0.88) | 0.82 (0.79, 0.87) | 0.66 (0.62, 0.70) | 0.73 (0.65, 0.80) | | | | | | |
| **SGA** | | | |  |  | | | | | | |
|  | Crude | 0.80 (0.76, 0.84) | 0.72 (0.70, 0.75) | 0.64 (0.62, 0.67) | 0.69 (0.64, 0.75) | | | | | | |
|  | Adjusted | 0.78 (0.74, 0.82) | 0.69 (0.67, 0.71) | 0.60 (0.57, 0.63) | 0.64 (0.59, 0.69) | | | | | | |
| Abbreviation: PTB: Preterm Birth = gestational age < 37 weeks; LBW: Low Birth Weight = birth weight < 2.5 kg; SGA: Small for Gestational Age = birth weight below 10th centile for gestational age. | | | | | | | | | |  |  |
|  |  |  |  |  |  |  |  |  |  |  |  |
| Reference: Nulliparity | | | | | |  |  |  |  |  |  |
| Adjusted for maternal race, residence, immigrant, education, pre-pregnancy obesity, paternal age and race, sex of newborn. | | | | | | | | | |  |  |
