# Supplementary material for: Parity and the risks of adverse birth outcomes: a retrospective study among Chinese
Source: BMC Pregnancy Childbirth. 2021 Mar 26;21:257. doi: 10.1186/s12884-021-03718-4 (PMC8004392; doi:10.1186/s12884-021-03718-4)
Supplement: Supplementary file 3 — Additional file 3: Table S3. Relative Risk (RR) for adverse birth outcomes by parity, among overall women and subgroups with missing data imputed. [file 12884_2021_3718_MOESM3_ESM.docx]

**Parity and the risks of adverse birth outcomes: a retrospective study among Chinese**

Li Lin^1^, Ciyong Lu^1^, Weiqing Chen^1^, Chunrong Li ^2*^, Vivian Yawei Guo^1*^

^1^ Department of Epidemiology, School of Public Health, Sun Yat-sen University, Guangzhou, Guangdong, China

^2^ Chengdu Women's and Children's Central Hospital, School of Medicine, University of Electronic Science and Technology of China, Chengdu, Sichuan, China.

* Corresponding authors:

Chunrong Li,

Chengdu Women's and Children's Central Hospital, School of Medicine, University of Electronic Science and Technology of China, Chengdu, Sichuan, 611731, China. Email: [cdlcr@163.com](mailto:cdlcr@163.com)

Vivian Yawei Guo,

Department of Epidemiology, School of Public Health, Sun Yat-sen University, Guangzhou, Guangdong, 510080, China. Email: [guoyw23@mail.sysu.edu.cn](mailto:guoyw23@mail.sysu.edu.cn)

| **Table S3** Relative Risk (RR) for adverse birth outcomes by parity, among overall women and subgroups with missing data imputed | | | |  |
| --- | --- | --- | --- | --- |
|  |  | **Crude RR (95% CI)** | **Adjusted RR (95% CI)** |  |
|  |  |  |  |  |
| **PTB** | | | |  |
|  | Overall | 1.07 (1.05, 1.09) | 0.87 (0.85, 0.89) |  |
|  | MPTB | 1.09 (1.07, 1.11) | 0.89 (0.87, 0.91) |  |
|  | VPTB | 1.18 (1.06, 1.33) | 0.95 (0.82, 1.10) |  |
| **LBW** | | | |  |
|  | Overall | 0.92 (0.90, 0.94) | 0.77 (0.74, 0.79) |  |
|  | Preterm birth | 0.86 (0.83, 0.89) | 0.84 (0.81, 0.88) |  |
|  | Term birth | 0.72 (0.69, 0.74) | 0.64 (0.61, 0.67) |  |
| **SGA** | | | |  |
|  | Overall | 0.66 (0.64, 0.67) | 0.65 (0.64, 0.67) |  |
|  | Preterm birth | 0.67 (0.63, 0.72) | 0.59 (0.55, 0.64) |  |
|  | Term birth | 0.65 (0.64, 0.67) | 0.66 (0.65, 0.68) |  |
| Abbreviation: PTB: Preterm Birth; LBW: Low Birth Weight; SGA: Small for Gestational Age; MPTB: Moderate Preterm Birth; VPTB: Very Preterm Birth. | | | |  |
|  |  |  |  |  |
| MPTB was defined as gestational age between 32-36 weeks; VPTB was defined as gestational age between < 32 weeks; PTB was defined as gestational age < 37 weeks, LBW was defined as birth weight < 2500 g; SGA was defined as birth weight below 10th centile for specific gestational age and sex. | | | |  |
| Reference group: Nulliparity | | | |  |
| Adjusted for maternal age and race, residence, immigrant, education, pre-pregnancy obesity, paternal age and race, sex of newborn. | | | |  |
