# Supplementary material for: Parity and the risks of adverse birth outcomes: a retrospective study among Chinese
Source: BMC Pregnancy Childbirth. 2021 Mar 26;21:257. doi: 10.1186/s12884-021-03718-4 (PMC8004392; doi:10.1186/s12884-021-03718-4)
Supplement: Supplementary file 4 — Additional file 4: Table S4. Associations between parity/maternal age and adverse birth outcomes with missing data imputed (adjusted RR and 95% CI). [file 12884_2021_3718_MOESM4_ESM.docx]

**Parity and the risks of adverse birth outcomes: a retrospective study among Chinese**

Li Lin^1^, Ciyong Lu^1^, Weiqing Chen^1^, Chunrong Li ^2*^, Vivian Yawei Guo^1*^

^1^ Department of Epidemiology, School of Public Health, Sun Yat-sen University, Guangzhou, Guangdong, China

^2^ Chengdu Women's and Children's Central Hospital, School of Medicine, University of Electronic Science and Technology of China, Chengdu, Sichuan, China.

* Corresponding authors:

Chunrong Li,

Chengdu Women's and Children's Central Hospital, School of Medicine, University of Electronic Science and Technology of China, Chengdu, Sichuan, 611731, China. Email: [cdlcr@163.com](mailto:cdlcr@163.com)

Vivian Yawei Guo,

Department of Epidemiology, School of Public Health, Sun Yat-sen University, Guangzhou, Guangdong, 510080, China. Email: [guoyw23@mail.sysu.edu.cn](mailto:guoyw23@mail.sysu.edu.cn)

| **Table S4** Associations between parity/maternal age and adverse birth outcomes with missing data imputed (adjusted RR and 95% CI) | | | | | |
| --- | --- | --- | --- | --- | --- |
|  | | **Maternal age (y)** | | | |
|  |  | **<25** | **25-29** | **30-34** | **≥35** |
| **PTB** | | | |  |  |
|  | Nulliparity | 0.98 (0.95, 1.01) | 1 (ref) | 1.19 (1.15, 1.24) | 1.57 (1.47, 1.69) |
|  | Multiparity | 0.95 (0.90, 1.01) | 0.95 (0.92, 0.98) | 1.06 (1.02, 1.09) | 1.42 (1.36, 1.48) |
| **LBW** | | | |  |  |
|  | Nulliparity | 1.08 (1.04, 1.12) | 1 (ref) | 1.24 (1.18, 1.29) | 1.46 (1.34, 1.60) |
|  | Multiparity | 0.86 (0.81, 0.92) | 0.79 (0.76, 0.82) | 0.86 (0.82, 0.89) | 1.18 (1.12, 1.24) |
| **SGA** | | | |  |  |
|  | Nulliparity | 1.14 (1.11, 1.17) | 1 (ref) | 1.03 (1.00, 1.07) | 1.11 (1.04, 1.20) |
|  | Multiparity | 0.84 (0.80, 0.88) | 0.65 (0.64, 0.67) | 0.62 (0.60, 0.64) | 0.75 (0.72, 0.78) |
| Abbreviation: PTB: Preterm Birth; LBW: Low Birth Weight; SGA: Small for Gestational Age. | | | | | |
| PTB was defined as gestational age < 37 weeks, LBW was defined as birth weight < 2500 g; SGA was defined as birth weight below 10th centile for specific gestational age and sex. | | | | | |
| Reference group: Nulliparity/aged between 25-29 years. | | | | | |
| Adjusted for maternal race, area of residence, immigrant status, education, pre-pregnancy obesity, paternal age and race, and sex of newborn. | | | | | |
